# Low-annotation apple flower counting: A color-SAM enhanced and uncertainty-guided semi-supervised framework

**DOI:** 10.1016/j.plaphe.2026.100190

**Published:** 2026-03-03

**Authors:** Yu Wang, Xuchao Guo, Jingzhong Huang, Chengyu Chen, Xiangfei Zhuge, Wen Chu, Xia Hao

**Affiliations:** College of Information Science and Engineering, Shandong Agricultural University, N0. 61, Daizong Road, Taian, 271018, Shandong Province, China

**Keywords:** Flower counting, Density estimation, SAM, Semi-supervised learning, Uncertainty

## Abstract

Accurate flower-load assessment is critical for informed thinning strategies in orchard management. UAV-based deep learning automated counting offers efficiency advantages, yet precise counting is heavily dependent on abundant annotated data, which is scarce and costly to obtain in agricultural settings. While semi-supervised learning alleviates dependency on manual annotation, its application to UAV-based orchard imagery faces challenges: complex backgrounds and small target sizes, which undermine pseudo-label reliability. To address these challenges, this study proposes a two-stage framework to achieve separate counting of apple flowers at different phenological stages. First, a color-SAM flower extractor (CSAM-FE) is proposed to preprocess images using a strategy combining color thresholding with the Segment Anything Model (SAM), suppressing background noise and extracting high-quality flower clusters, thereby providing purified inputs for the subsequent counting network. Second, an uncertainty-guided semi-supervised flower counting network (USCount-Net) is proposed for accurate stage-specific flower counting with limited labeled data. The USCount-Net incorporates two key components: an adaptive pseudo-label filtering (PLF) mechanism based on frequent forward uncertainty estimation (FFUE) is designed to dynamically suppress noisy gradient backpropagation, mitigating error propagation from unreliable pseudo-labels; and a noise-sensitive adaptive gated fusion (AGF) module is introduced to fuse cross-scale features without redundancy, addressing significant scale variations across phenological stages and observation angles. Comparative experiments on a self-built apple flower counting dataset demonstrate that USCount-Net achieves lower MAE and RMSE than state-of-the-art methods at 10%, 30%, and 50% labeling ratios. The results demonstrate that the proposed methodology serves as methodological support for rapid and precise apple flower counting in low-annotation agricultural scenarios.

## 1 Introduction

Ensuring apple fruit quality attributes such as size, coloration, and sugar content, while maintaining high orchard productivity, necessitates the common grower practice of flower thinning. This intervention enhances resource allocation to the retained flowers, improving subsequent fruit development. However, this procedure is best executed during the full bloom stage, requiring strategies calibrated to the specific floral intensity and spatial distribution exhibited by individual trees [1]. Consequently, the timely and precise quantification of apple flower abundance constitutes a critical prerequisite. It provides the fundamental basis for estimating orchard crop load, predicting the peak flowering period and formulating targeted flower thinning strategies.

Traditional manual sampling methods are time-intensive and susceptible to observer variability, limiting their effectiveness for precision orchard management. Computer vision-based phenotype analysis offers an efficient solution for optimizing the intelligent construction of orchards by quantitative analysis of flower density, spatial distribution, and developmental stages. Early research leveraged the distinct color contrast between flowers and background, utilizing color threshold segmentation to isolate flower objects [2]. However, the imaging of apple flowers in field conditions is susceptible to variations in lighting and angle, and traditional manual feature extraction methods are sensitive to these factors, making them unable to meet the demand for accurate flower counting.

Recently, advances in deep learning for image analysis have revolutionized computer vision applications in plant phenotyping. These techniques are now widely adopted for automated analysis of key phenotypic traits including plant height measurements [3], leaf angle measurements [4], and leaf vein segmentation [5]. Automated agricultural object counting, a fundamental component of precision agriculture, has been successfully implemented for critical applications such as crop counting [[6], [7], [8]], critical growth trait analysis [7]. Given its critical role in orchard automation, automated flower counting in fruit trees has attracted increasing research attention. Widely adopted object detection algorithms such as the YOLO series and Faster R-CNN [9] are now applied to monitor bloom stages and localize flowers. This facilitates quantitative assessment of regional bloom density [10] and provides essential algorithmic support for the vision system of the flower thinning robot [11]. However, these methods are primarily designed for close-range, side-view imaging scenarios. When applied to high-density orchard-scale apple flower counting, detection accuracy decreases exponentially with increasing target density. Their complex network architectures also struggle to meet real-time processing requirements. Recently, UAVs have demonstrated significant potential for high-throughput orchard phenotyping due to their aerial perspective, broad field of view, and efficient data collection capabilities. This enables novel pathways for smart farming initiatives and assisted breeding programs. Nevertheless, the wide observational scope introduces new challenges: heightened background complexity, enormous target quantity, and dense occlusion.

Density estimation methods implicitly model spatial distributions by mapping input images to continuous density maps, as evidenced by their successful applications in counting tasks [12]. By embedding high-density target information into the global density distribution, these methods effectively circumvent challenges posed by mutual occlusion between densely packed targets. This characteristic makes them particularly suitable for high-density, occlusion-prone in situ crop counting scenarios under UAV-based imaging. To enhance robustness in complex agricultural environments and adaptability to specific crop phenotypic traits, various improved density estimation frameworks have been proposed. SPSC-Net [13] integrates an enhanced YOLOX model into a two-stage canopy localization-shoot counting architecture to addresses the complex canopy backgrounds and dense, tiny shoots characteristic of slash pine, enabling automated shoot counting. FCNet [14] introduces a counting information enhancement module and interference loss to separate domain-specific features, enabling robust fish counting in turbid and low-light underwater environments. MCPCNet [6] leverages semantic enhancement and scale adaptation modules to count multiple crop species with significant morphological variations from UAV images. In the field of in-situ flower counting, Density-Cluster-Count [15], FlowerNet [16] and a comparative study on flower counting between detection and density estimation method [17] have empirically verified the effectiveness of density estimation for dense flower clusters.

However, as illustrated in Fig. S1, direct density estimation of apple flowers from UAV imagery faces several formidable challenges: primarily, severe background noise and the inherently small size of the targets; furthermore, their distribution is locally dense yet globally sparse, and the overlapping of flowers at different phenological stages within local clusters frequently leads to false and missed detections.

To address the above difficulties, two primary mitigation strategies have emerged: (1) Multi-task learning approaches incorporate segmentation branches into network backends [16] to enable foreground-background discrimination to suppress non-target region interference, and ultimately enhancing the model's focus on the target regions; (2) Color-feature-based preprocessing methods, which utilize predefined flower color thresholds to extract flower region pixels, providing background-free inputs for density estimation networks [8,17], or directly utilize threshold segmentation masks to quantify tree-level blooming intensity [18]. While these solutions substantially mitigate background interference, reduce false/missed detections, and improve counting accuracy, they present inherent limitations: the former increases model complexity and requires additional annotated data for auxiliary branches, while the latter relies on manually designed features with limited robustness.

Furthermore, the high-precision counting performance of existing deep learning models relies on large-scale annotated datasets. However, annotation scarcity in agricultural scenario severely limits their practical implementation. Expanding datasets under constrained labeling cost has thus become an urgent requirement for enhancing model robustness.

To alleviate dependence on massive high-quality annotations, semi-supervised learning (SSL) methods have been explored. Among these, the Mean-Teacher framework [19] employs parallel teacher-student models with shared architectures and parameters, where a teacher model generates pseudo-labels for unlabeled samples that serve as supervisory signals for student model unlabeled training, thereby guiding the model to enhance its generalization from unlabeled samples. Subsequent Studies based on this framework have primarily focused on data augmentation. Specifically, by enforcing consistency between teacher-student models under varying perturbation intensities of the same unlabeled sample to optimize the model. The researchers have experimented with various strategies such as color transformation, scale jittering, random flipping, and geometric transformation [20], and even introduced gradient perturbations through virtual adversarial training to more precisely measure local prediction smoothness [21], thereby enhancing performance in dense scenarios.

Nevertheless, domain-specific noise in agriculture (e.g., uneven illumination, foliar occlusion, background clutter) degrades pseudo-label quality. Directly conducting consistency constraints using unrefined pseudo-labels risks propagating noise and diverting the model from optimal solutions. Notably, our prior work [22] demonstrated that auxiliary information effectively guides model training and reducing interference from irrelevant data. Building on this insight, this study aims to develop a method to quantify pseudo-label reliability through a control mechanism. In semi-supervised classification tasks, the model leverages class probabilities as confidence metrics for pseudo-label quality [23]. In contrast, regression-based density estimation lacks analogous confidence estimation mechanisms, making pseudo-label filtering fundamentally more challenging and preventing direct transfer of traditional approaches.

Uncertainty estimation offers a promising solution for pseudo-label quality assessment. While this method has achieved success in semi-supervised image segmentation [[24], [25], [26], [27]], object detection [28,29], and affect recognition [30], its adaptation to dense agricultural counting tasks remains unexplored territory.

To address the critical issues of dense flower distributions, complex backgrounds, and limited high-quality annotations in UAV imagery, in this paper, we propose the uncertainty-guided semi-supervised flower counting network (USCount-Net). It enables apple flower counting under low annotation cost by designing a density estimation framework integrated with uncertainty quantification. The main contributions of this study are as follows:

(1) An uncertainty-guided pseudo-label filtering mechanism designed for regression-based density estimation is proposed to significantly enhances the robustness of semi-supervised flower counting under complex field conditions.
(2) A training-free CSAM-FE preprocessing module is proposed to isolate flower cluster pixels. This strategy combines color-thresholding with the Segment Anything Model [31] for high-quality flower cluster extraction, effectively purifying inputs and reducing data volume.
(3) A noise-sensitive AGF module is introduced to dynamically weights multi-scale features to preserve petal texture details while enhancing flower cluster distribution semantics via a learnable spatial gating mask. This module suppresses noise from redundant features through adaptive fusion.

The following sections systematically detail the proposed methodology. Section 2 presents a comprehensive overview of the dataset construction pipeline, and the detailed architecture of CSAM-FE and USCount-Net. Section 3 subsequently demonstrates the model performance through comparative experiments, ablation studies, and quantitative uncertainty analysis conducted on the self-built apple flower dataset. Section 4 further analyzes the proposed methodology by critically examining its technical advantages, inherent limitations, and potential research extensions. Finally, Section 5 summarizes the key contributions and outcomes of this study, highlighting its significance in advancing semi-supervised counting methodologies for agricultural applications.

## 2 Materials and methods

### 2.1 Construction of the apple flower counting dataset

#### 2.1.1 Data acquisition

**Fig. 1:**
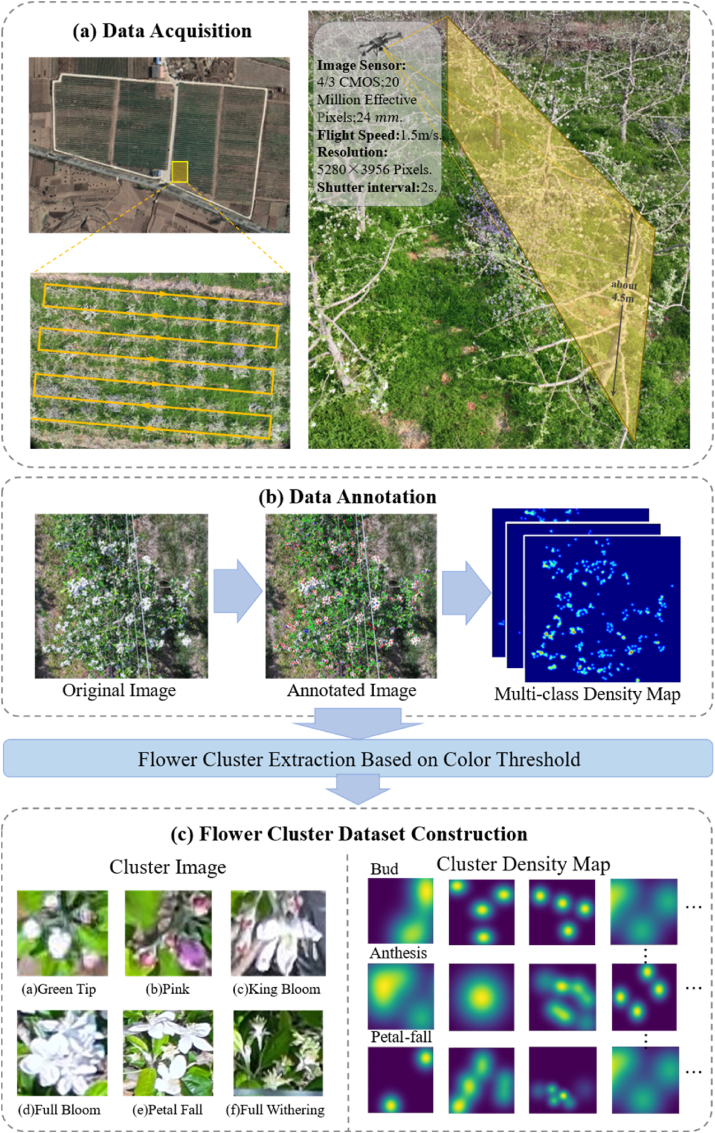
Apple flower counting dataset construction pipeline. (a) Data acquisition conditions and UAV photography parameters. (b) Example of data annotation pipeline. (c) Final dataset with flower cluster images and density map of three phenological stages.

This study focused on three apple cultivars, i.e., Gala, Golden Delicious, and Fuji, two rootstock types, namely dwarfing and standard, covering three developmental stages of the tree, including initial bloom, full bloom, and late bloom. As shown in Fig. 1(a), images were collected from the China-Israel Modern Agricultural Technology Demonstration Park in Yiyuan County, Zibo City, Shandong Province, China (36°12′N, 118°07′E). A DJI Mavic 3 Pro UAV equipped with a 24 mm equivalent focal length camera (5280 × 3956 pixels resolution) was utilized for image acquisition. Data collection occurred on three dates: April 12, 2024, April 20, 2024, and April 10, 2025, during the time window of 07:00–12:00. The UAV followed a zigzag flight path at 7 m altitude (≈ 2.5 m above the canopy) and captured images vertically downward via hovering at fixed points, with the pitch angle was adjusted ± 5° according to tree growth patterns. To ensure consistent image quality, all acquisitions were conducted on the sunlit side of the orchard.

Given the constraint of the practical infeasibility of exhaustive flower capture, our imaging protocol design is underpinned by two principles. First, standardized nadir imaging ensures data consistency across samples, creating an equitable basis for model training and comparison. Second, canopy-level flower metrics are established as a reliable proxy for whole-tree bloom status, justified by the strong correlation between canopy samples and integral flowering characteristics [32,33]. This standardized canopy-based quantitative approach adequately serves the research objectives of this study.

#### 2.1.2 Dataset construction

After quality screening, 332 apple tree images were selected for apple flower counting research: 126 images at initial bloom, 150 at full bloom, and 56 at late bloom stage. Given the complexity of apple tree growth, where at the same growth stage, usually contains apple flowers from multiple phenological stage, quantifying floral distribution patterns across phenological stages provides critical insights for orchard management. Therefore, under expert guidance, flowers were annotated as three classes: bud, anthesis, and petal-fall, using point annotations. As shown in Fig. 1(b), bud annotations were placed at geometric centers, while anthesis and petal-fall annotations were positioned at floral pistils. The number of annotations per tree image varies by its developmental stage: 50–150 in the initial bloom stage, 100–200 in the full bloom stage, and 30–100 in the late bloom stage.

Subsequently, we employed a geometry-adaptive kernel method [34] to map point annotations into density maps. These maps served as intermediate representations for supervising a counting model, effectively handling complex scenes with dense canopy overlaps. Fig. 1(b) exemplifies resulting density maps, where dark blue indicates background and brighter hues denote regions of densely clustered flowers.

#### 2.1.3 Color-SAM flower cluster extraction preprocessing

In UAV-based apple flower counting, small-scale flowers are often submerged by complex background noise, posing significant challenges for accurate and efficient counting, particularly given the high resolution of raw UAV images. To address these issues, this study proposes an innovative color-SAM flower extractor (CSAM-FE) designed to suppress background interference, highlight flower cluster regions, and enhance the accuracy and efficiency of subsequent counting models.

This module leverages Segment Anything Model (SAM) [31], a pre-trained, large-scale segmentation model with robust zero-shot generalization capability to achieve high-precision segmentation without task-specific fine-tuning. Since SAM's performance critically depends on effective prompt inputs, we automatically extract floral region pixels via color thresholding, converting them into point prompts to guide SAM's target localization. By combining color-threshold segmentation with SAM for refined segmentation, the proposed method substantially reduces dependency on handcrafted features (e.g., meticulously tuned RGB thresholds). Notably, as a training-free and independent preprocessing step, CSAM-FE avoids the complexity and additional annotation burden associated with designing multi-task architectures, ensuring computational efficiency and practical scalability.

**Fig. 2:**
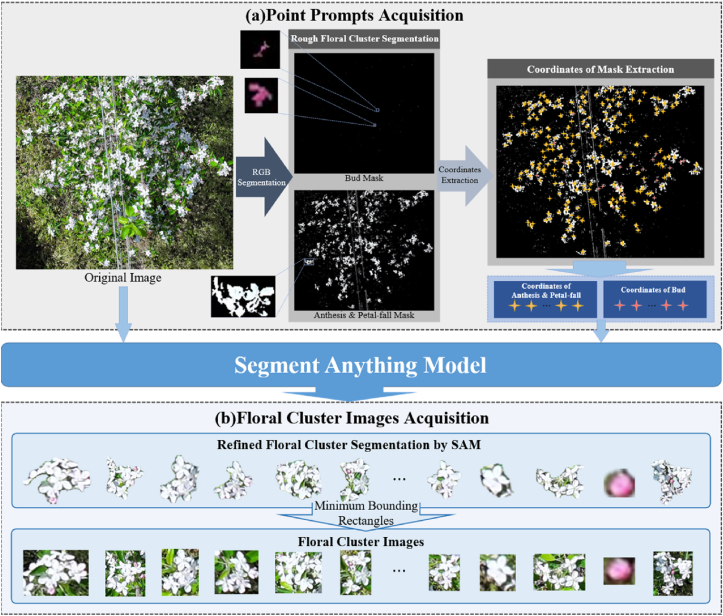
Flower cluster extraction pipeline. (a) Flower cluster pixel extraction via color thresholding, where the centroid of each connected region in the flower cluster mask serves as a point prompt for SAM. The prompts are marked by four-pointed stars in the rightmost image of (a): yellow markers indicate anthesis and petal-fall stages, while pink markers denote bud-stage clusters. (b) The refined segmentation results by SAM and the final flower cluster images after minimum bounding rectangle processing.

First, as shown in Fig. 2(a), leveraging the significant color difference between flower and background, a strict RGB color thresholding is applied to raw UAV orchard images for rough flower cluster segmentation. The color thresholds were empirically determined to maximize the segmentation accuracy on our dataset. Specially, this selection process was based on a statistical analysis of the color distribution in manually annotated regions of interest (ROIs). We observed that buds, due to their reddish hue, exhibit high Red and low Green values in the RGB color space, which are distinguished from the background. While anthesis or petal-fall flowers appear almost white under illumination, leading to high values across all channels. However, many background elements also exhibit high values in the Red and Green channels. We capitalize on the superior contrast in the Blue channel to isolate targets by applying a high Blue threshold. These specific numerical values were optimized by evaluating the F1-score of pixel-wise segmentation against a validation set. The stage-specific threshold values are as follows:

(1) $$\left\{ \begin{array}{c} Red>120⋂Green<100,bud\\ Blue>220,anthesis\;and\;petal-fall \end{array}\right.$$

Employing a strict threshold aims to maximize background noise suppression, preventing misleading point prompts for SAM rather than preserving complete floral features. This inevitably caused partial loss of floral pixels (as illustrated in Fig. 2(a)). Subsequently, the centroid coordinates of each connected region from the preliminary segmentation are computed and input as point prompts to SAM. As shown in Fig. 2(b), the original image and the generated point prompts are simultaneously fed into the pre-trained SAM model. Leveraging SAM's robust zero-shot generalization capability, the model performs refined flower cluster segmentation, producing precise flower cluster masks.

Then, minimum bounding rectangles are computed for each refined floral mask output by SAM. These rectangular regions are cropped to generate the final flower cluster images. These rectangular regions were cropped to generate final flower cluster images. The adoption of minimum bounding rectangles instead of irregular masks is primarily motivated by two considerations: (1) Providing essential spatial contextual information: When flowers are partially occluded by leaves, the minimal background preserved within rectangular regions assists subsequent feature extraction models in comprehending local contexts and inferring occluded structures; (2) Enhancing segmentation robustness: Rectangular boundaries ensure regular geometry and target integrity, preventing edge information loss during cropping and optimizing compatibility with standard convolutional neural networks.

Finally, after CSAM-FE processing, a significant reduction in the number of processed pixels is achieved, with a minimum of >80% and an average of 98.76%, determined by the pixel comparison between original orchard images and their extracted flower clusters. This significantly increases computational efficiency while preserving critical spatial information. The extracted 9042 flower cluster images are randomly partitioned into training, validation, and test sets at a 7:2:1 ratio as inputs to the counting network. Fig. 1(c) presents examples of flower cluster images and density maps across phenological stages.

### 2.2 Design of uncertainty-guided semi-supervised apple flower counting model

**Fig. 3:**
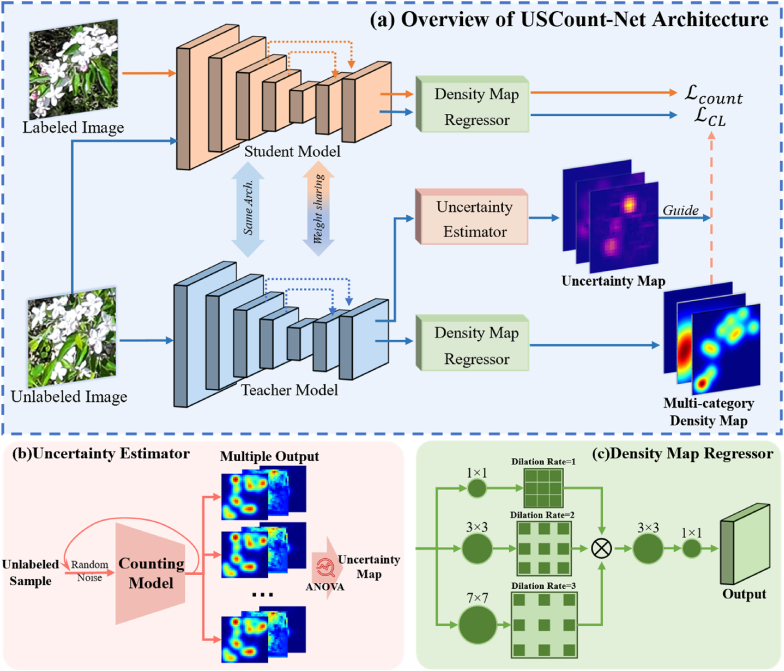
Overall architecture of USCount-Net. (a) Overall architecture of USCount-Net, comprising a parallel teacher-student model. (b) Uncertainty estimation through variance analysis via multi-round forward propagation. (c) Density map regressor with multi-scale dilated convolution branches: 1 × 1, 3 × 3, and 7 × 7 convolutions followed by dilated convolutions with dilation rates of 1, 2, and 3, respectively.

To achieve accurate apple flower counting across phenological stages with minimal annotation costs, this study proposes USCount-Net. As illustrated in Fig. 3, the model features a symmetric dual-model architecture based on the Mean Teacher framework [19]. Both models share four core components: (1) A modified VGG-19 [35] backbone network for foundational feature extraction; (2) An AGF module dynamically merges extracted multi-scale feature, allowing the model to maintain local accuracy while understanding global structure, thus improving counting performance across varying flower sizes and occlusion conditions; (3) A convolutional block attention module (CBAM) [36] modeling channel-spatial dependencies to focus on flower regions while suppressing background interference; (4) A density map regressor (DMR) employing multi-scale dilated convolutions [37] to extract multi-resolution flower features, subsequently mapping optimized features to high-quality density maps across phenological stages (i.e., the final output). The teacher model exclusively incorporates the FFUE module, activated only during unsupervised training. Crucially, FFUE performs pixel-level uncertainty quantification for pseudo-labels generated from unlabeled samples, providing a quantitative basis for evaluating pseudo-label quality.

During the labeled training phase, only the student model is activated and optimized via the counting loss $L_{Count}$, with ground truth density maps as supervisory signals. Concurrently, teacher model parameters are updated through exponential moving average (EMA) synchronization with student model weights. In the unlabeled training phase, the student-teacher models are jointly trained. The teacher model generates pseudo-labels and corresponding uncertainty maps via DMR and FFUE module. These pseudo-labels, filtered by an uncertainty-driven pseudo-label filtering (PLF) mechanism, serve as supervision signals for the student model. This phase is optimized using the uncertainty-guided consistency loss $L_{CL}$.

In summary, USCount-Net enhances robustness to scale variations across phenological stages and complex background interference, ultimately generating density distribution maps and quantitative counts of apple flowers across different phenological stages, thereby providing essential technical support for smart orchard management. The subsequent sections provide detailed design and functionality of each module.

#### 2.2.1 Feature fusion

**Fig. 4:**
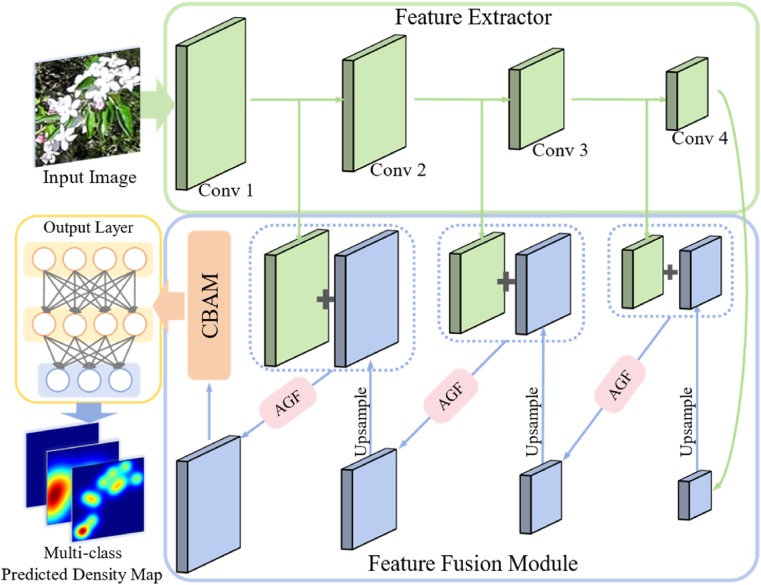
Feature extraction and fusion networks.

To achieve efficient feature representation for flower cluster images, as illustrated in Fig. 4, the proposed model employs the first 13 convolutional layers of VGG-19 pre-trained on ImageNe as the backbone network for feature extraction. To reduce model complexity, the fifth convolutional block and all subsequent pooling and fully-connected layers of the VGG-19 network were removed, retaining only the first four convolutional blocks (Conv1-Conv4).

As illustrated in Fig. 4, an input image with dimensions H×W×3 undergoes hierarchical feature extraction through these four convolutional blocks, sequentially producing multi-scale feature representations. These outputs consist of four feature maps with dimensions H×W×64, $\frac{H}{2}\times \frac{W}{2}\times 128$, $\frac{H}{4}\times \frac{W}{4}\times 256$, $\frac{H}{8}\times \frac{W}{8}\times 512$, respectively.

#### 2.2.2 Noise-sensitive adaptive gated feature fusion module

Based on the analysis of feature hierarchy characteristics, feature maps from shallow layers contain rich detailed information, while deeper feature maps focus more on semantic representation. By integrating high-resolution features extracted from shallow networks with high-level semantic features from deep networks, multi-scale information can be captured, effectively preserves fine details, and accelerates model convergence. Building upon this principle, we construct an adaptive gated feature fusion (AGF) module**.** As shown in Fig. 4, this module progressively fuses deep features carrying abstract information (such as floral organ semantic categories) with shallow features extracted from the first three VGG blocks (Conv1-Conv3). This strategy simultaneously preserves high-precision details (e.g., edge textures, petal contours) while enhancing semantic expressiveness.

**Fig. 5:**
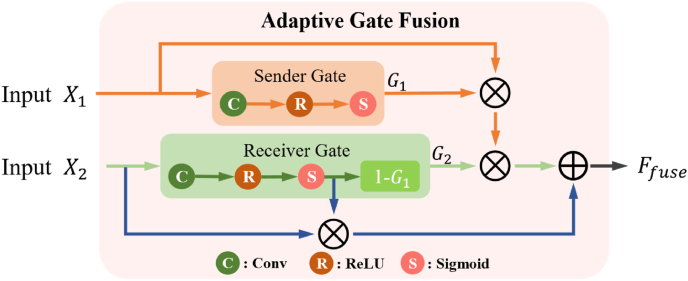
Detailed architecture of adaptive gated fusion module.

The fusion process follows a top-down pathway with lateral connections: deep features are first upsampled to match the resolution of shallow features via bilinear interpolation, followed by 1×1 convolution to align channel dimensions before cross-level feature integration. However, conventional multi-level feature fusion methods, such as concatenation, element-wise addition, or Feature Pyramid Networks [38], fuse features from all hierarchical levels indiscriminately. This approach lacks adaptive differentiation of feature importance and may introduce redundant information. In contrast, the proposed AGF module, as shown in Fig. 5, dynamically learns feature importance through gated units, enabling the selective aggregation of cross-level information.

Specifically, the AGF module consists of a sender gate for the feature to be fused and a receiver gate for the primary feature. These gates adaptively generate gate maps that implicitly model feature noise probability distributions to localize noisy regions via a 3 × 3 convolutional layer, computed as follows:

(2) $$F_{i}=ReLU\left(\begin{array}{c} {Conv}_{3\times 3}\left(X_{i}\right) \end{array}\right)$$

where $X_{i}$ denotes the input to the i-th feature layer. Subsequently, a 1×1 convolution and Sigmoid function produce a spatial gate map $G_{i}$ reflecting feature reliability:

(3) $$G_{i}=\sigma \left(\begin{array}{c} {Conv}_{1\times 1}\left(F_{i}\right) \end{array}\right)$$

where $G_{i}$ denotes the spatial gate map of $X_{i}$, where its values (normalized within [0,1]) represent the reliability of the corresponding feature regions. Higher values indicate higher confidence in the feature validity.

Through the gate map processing, suppressive weighting is applied to noisy regions, preserving only high-response effective features exceeding the threshold. Subsequently, the receiver gate of the AGF performs reverse filtering ($1-G_{a}$) on the sender gate, suppressing redundant information while supplementing missing discriminative features.

Finally, the complementary information refined through dual gating is weighted and fused to obtain the redundancy-reduced fusion feature $F_{fuse}$:

(4) $$F_{fuse}=\left(X_{1}⊗G_{1}\right)⊕\left(1-G_{1}\right)⊗\left(X_{2}⊗G_{2}\right)$$

where ⊗ denotes matrix multiplication, and ⊕ denotes element-wise addition, $X_{1}$ and $X_{2}$ represent the features to be fused, while $G_{1}$ and $G_{2}$ denote their corresponding spatial gate maps. By implementing this dynamic gated feature fusion mechanism, high-quality fused features are ultimately obtained. This mechanism effectively utilizes the detailed information from shallow features and the semantic information from deep features while adaptively handling noise.

#### 2.2.3 Adaptive pseudo-label filtering mechanism based on frequent forward uncertainty estimation

As illustrated in Fig. 3, during the unlabeled training phase, we construct a dual-path teacher-student feature alignment framework. In this framework, the student network generates density prediction distributions from strongly augmented images, while the teacher network produces pseudo-labels as supervisory signals based on weakly augmented images. A consistency loss $L_{CL}$ enforces output distribution consistency between the two pathways. This approach compels the model to maintain prediction stability under data perturbations, thereby effectively capturing the latent feature representations within unlabeled samples.

However, in natural field scenarios, severe noise caused by uneven illumination and leaf occlusion can significantly degrade the quality of pseudo-labels generated by the teacher network. To address these challenges, we propose an uncertainty-guided pseudo-label filter (PLF) mechanism. This approach dynamically masks low-confidence pixels in pseudo-labels, thereby mitigating model degradation caused by unstable pseudo-label quality in traditional semi-supervised methods.

The PLF mechanism comprises an uncertainty estimation module and an adaptive spatial consistency loss mask. First, a pixel-wise frequent forward uncertainty estimation (FFUE) is developed based on multiple forward passes, generating a spatial confidence map through variance measurement. Specifically, the same model performs T independent forward passes on identical samples with varying perturbation levels as input. The variance $U_{var}\left(x\right)$ of predicted density values is then computed to quantify the model's uncertainty for the sample. The mathematical formulation of $U_{var}\left(x\right)$ is as follows:

(5) $$U_{var}\left(x\right)=\sqrt{\frac{1}{T}\sum_{t=1}^{T}{\left(D_{t}\left(x\right)-\bar{D}\left(x\right)\right)}^{2}}$$

where $D_{t}\left(x\right)$ represents the predicted value at coordinate x in the t-th forward sampling, and $\bar{D}\left(x\right)$) represents the mean of the predicted values from T samplings.

Subsequently, an adaptive loss mask mechanism is proposed, building upon the pixel-wise uncertainty metric $U_{var}\left(x\right)$. Specifically, the average uncertainty index calculated across all spatial locations of a pseudo-label, quantifies the global confidence in the model prediction. This value dynamically eliminates the consistency loss during the unlabeled training phase. Given the structural diversity of apple flowers across phenological stages, predictions inevitably encounter heterogeneous noise patterns. To address this, we propose channel-specific uncertainty thresholds that adaptively adjust to the noise characteristics of each feature channel. The adaptive threshold $t_{k}$ for channel k is calculated as follows:

(6) $$t_{k}=\frac{1}{W\cdot H}\sum_{i=1}^{W}\sum_{j=1}^{H}{U_{var}\left(x\right)}_{i,j,k}$$

where, ${U_{var}\left(x\right)}_{i,j,k}$ denotes the uncertainty value at spatial coordinates (i,j) in channel k of the predicted density map. As the model accuracy improves, the pixel-wise prediction reliability increases, causing the threshold $t_{k}$ to decrease progressively during training. This mechanism effectively balances the trade-off between incorporating new samples and maintaining model stability, enabling the model to assimilate fresh information while preventing performance degradation caused by noise or inaccurate pseudo-labels. Consequently, the model achieves continuous performance enhancement in the flower counting task.

Subsequently, the loss mask M is constructed based on $t_{k}$. If the uncertainty value of a pixel is below $t_{k}$, its corresponding loss mask is set to 0; otherwise, it is assigned 1. The mask value $M_{i,j,k}$ for the pixel at coordinates (i,j) in channel k is formulated as follows:

(7) $$M_{i,j,k}=\left\{ \begin{array}{cc} 1, & if\;{\;U_{var}\left(x\right)}_{i,j,k}>t_{k}\\ 0, & otherwise. \end{array}\right.$$

Then, in the process of constraining the consistency of the teacher-student model, the pixels higher than $t_{k}$ are eliminated via M, forcing the model only to focus on regions with low uncertainty for training. Specifically, for channel k, the pixel at coordinates (i,j) in the consistency loss $L_{CL}$ is masked out when its uncertainty value ${U_{var}\left(x\right)}_{i,j,k}$ falls below the threshold $t_{k}$. This masking process is computed as follows:

(8) $$L_{CL}^{M}=M⊙L_{CL}$$

where $L_{CL}^{M}$ represents the consistency loss after the M masking processing, and ⊙ represents element-by-element multiplication. When $M_{i,j,k}=0$, the corresponding position loss is masked; otherwise, it is unchanged.

#### 2.2.4 Loss function

The total loss function of USCount-Net $L_{total}$ comprises two components: (1) Counting loss $L_{Count}$ is applied during labeled training to refine the quality of density maps generated by the student model; (2) Consistency learning loss $L_{CL}$ is employed in unlabeled training to enforce prediction consistency between student and teacher models for unlabeled data to enhance model generalization capability. These losses balance supervised precision with unsupervised generalization under limited labeled data conditions. The formula of $L_{total}$ is expressed as follows:

(9) $$L_{total}=L_{Count}+L_{CL}$$

In Eq. (9), $L_{Count}$ combines the structural loss (SL) and $L_{1}$ loss to enhance density estimation accuracy through joint optimization:

(10) $$L_{Count}=SL\left(P,G\right)+\lambda L_{1}\left(P,G\right)$$

where P denotes the predicted density map, G represents the ground truth density map, and the weighting coefficient λ=0.01 is determined via prior knowledge [7]. SL employs a sliding window strategy to evaluate local discrepancies between predictions and ground truth. For pixel position (i,j), statistical measures are computed within a 3×3 local window:

(11) $$SL\left(P,G\right)=1-\frac{\left(2\mu_{P}\mu_{G}+C_{1}\right)\left(2\sigma_{PG}+C_{2}\right)}{\left(\mu_{P}^{2}+\mu_{G}^{2}+C_{1}\right)\left(\sigma_{P}^{2}+\sigma_{G}^{2}+C_{2}\right)}$$

where $\mu_{P}$ and $\mu_{G}$ denote the local means of P and G computed via Gaussian convolution. $\sigma_{P}^{2}$ and $\sigma_{G}^{2}$ represent the local variances, and $\sigma_{PG}$ is the local cross-covariance. The $L_{1}$ loss optimizes the regression accuracy of density maps by constraining the per-pixel absolute difference between the prediction and the ground truth:

(12) $$L_{1}\left(P,G\right)=\frac{1}{N}\sum_{i=1}^{N}\left|P_{i},G_{i}\right|$$

where $P_{i}$ and $G_{i}$ denote the i-th pixel values of the predicted and ground truth density maps respectively.

In Eq. (9), $L_{CL}$ facilitates collaborative optimization between student and teacher models by enforcing consistency constraints on unlabeled data. Specifically, weak and strong augmentations are applied to identical input samples, with $L_{CL}$ constraining prediction consistency between the two pathways. $L_{CL}$ is formalized as:

(13) $$L_{CL}=\frac{1}{N}\sum_{j=1}^{N}\left|P_{j}^{Stu},P_{j}^{Tea}\right|$$

where $P_{j}^{Stu}$ and $P_{j}^{Tea}$ represent the density maps predicted by the student network (processing weakly augmented samples) and teacher network (processing strongly augmented samples), respectively.

## 3 Results

### 3.1 Implement details and evaluation metrics

All experiments in this study were conducted using PyCharm 2022 and the PyTorch 1.13.1 deep learning framework [39], with specific experimental details provided in Table S1.

For performance evaluation metrics, we adopt the Mean Absolute Error (MAE) and Root Mean Square Error (RMSE) as fundamental evaluation metrics, which reflect the dispersion and robustness of prediction errors through squared penalties and absolute deviations, respectively. The calculation formulas are given in Eq. (14) and Eq. (15):

(14) $$MAE=\frac{1}{n}\sum_{i=1}^{n}\left|P_{i}-G_{i}\right|$$

(15) $$RMSE=\sqrt{{\frac{1}{n}\sum_{i=1}^{n}\left|P_{i}-G_{i}\right|}^{2}}$$

where $P_{i}$ denotes the predicted density map, and $G_{i}$ denotes the ground truth density map.

### 3.2 Comparison experiment

As shown in Table 1, comparative experiments were implemented to validate the effectiveness of the proposed counting methodology. It is important to note that the CSAM-FE is designed as a task-specific module. Therefore, we do not include direct comparisons with other segmentation methods. The effectiveness of CSAM-FE is validated by comparing the performance of counting models with versus without preprocessing (detailed in Section 3.3.1). The primary contribution of this work lies in the proposed counting model and its semi-supervised strategy. Consequently, the comparative analysis focuses specifically on the counting performance. To ensure experimental fairness and consistency, all comparative models, were trained and evaluated on the same dataset of flower cluster images preprocessed by CSAM-FE.

**Table 1:** Performance comparison with other counting models on the testing set.

Method	Model	Labeling Ratio	MAE			RMSE		
			Bud	Anthesis	Petal-Fall	Bud	Anthesis	Petal-Fall
Supervised	MCNN	100%	26.72	30.13	15.45	30.85	37.92	19.83
	CSR-Net		10.38	20.29	6.51	12.41	29.51	9.27
	DM-Count		6.23	7.34	5.18	10.85	10.76	8.47
	**USCount-Net**		**6.26**	**6.75**	**6.10**	**7.71**	**9.03**	**8.39**
Semi-Supervised	MT	10%	32.95	38.74	24.16	40.25	49.32	37.07
	IRAST		14.71	105.86	65.98	21.88	123.87	99.42
	Dream		27.69	38.75	20.47	39.58	51.94	27.43
	TreeFormer		24.35	33.61	9.31	37.41	45.71	12.87
	Calibrating		9.85	19.21	8.42	13.74	25.32	13.15
	**USCount-Net**		**7.59**	**11.21**	**6.88**	**8.89**	**17.49**	**8.89**
	MT	30%	30.48	35.77	22.66	42.77	45.58	35.83
	IRAST		11.41	111.35	69.92	17.41	129.02	17.41
	Dream		17.54	30.66	15.42	21.26	36.75	19.80
	TreeFormer		20.27	28.14	7.48	27.35	41.49	10.81
	Calibrating		10.39	13.21	9.75	13.87	18.76	11.72
	**USCount-Net**		**7.26**	**8.90**	**6.41**	**10.78**	**13.16**	**9.33**
	MT	50%	25.47	35.79	20.23	29.78	42.68	27.51
	IRAST		11.39	113.26	70.56	13.06	137.54	90.38
	Dream		13.35	16.21	7.22	20.55	27.76	10.65
	TreeFormer		17.93	26.78	6.83	25.86	39.07	8.47
	Calibrating		9.08	10.25	6.35	11.85	15.08	9.41
	**USCount-Net**		**7.08**	**8.93**	**6.61**	**10.46**	**13.41**	**8.32**

The evaluation was conducted across supervised and semi-supervised setting: first, comparisons were conducted against classical supervised counting models MCNN [40], DM-Count [41], CSRNet [42] to evaluate the applicability of the proposed architecture for apple flower counting tasks; second, comparisons with semi-supervised counting models MT [19], IRAST [43], Dream [44], TreeFormer [45], Calibrating [46] were performed to validate the effectiveness of the proposed semi-supervised learning strategy.

Table 1 presents the MAE and RMSE values for flower counting predictions across three phenological stages on the test set of our self-built apple flower counting dataset, comparing supervised and semi-supervised settings. USCount-Net demonstrates superior counting accuracy under both supervised (100% labeling ratio) and semi-supervised (10%, 30%, 50% labeling ratios) settings. Notably, at 30% and 50% labeling ratios, its accuracy approaches supervised performance.

While Calibrating, which also incorporates an uncertainty-aware mechanism, outperforms other semi-supervised counting models via improved pseudo-label quality, it exhibits performance degradation under low annotation ratios. This limitation stems from its reliance on post-processing calibration for uncertainty branch training rather than feature-level noise perception. Consequently, with severely limited annotated samples, the uncertainty branch suffers from inadequate training, leading to inaccurate predictions in uncertainty maps. IRAST shows substantial counting errors during the anthesis stage, attributable to its segmentation-based pseudo-label generation strategy designed for crowd counting, which is sensitive to boundary ambiguity in dense regions. This leads to misidentifying overlapping petals as distinct flowers, creating false peaks in density maps. Moreover, while Dream and TreeFormer demonstrate remarkable counting accuracy in crowd counting tasks, their performance on apple flower counting proves less optimal. We suppose this limitation is attributed to their respective mechanisms: Dream's rank-consistent pyramid loss and TreeFormer's transformer-based global attention excel at capturing global distribution patterns but exhibit insufficient sensitivity to local details (e.g., petal edges, small-target textures), resulting in confused feature representations in densely occluded regions.

In contrast, USCount-Net demonstrates significant robustness under low labeling ratios through synergistic optimization of its model architecture and semi-supervised strategy. Specifically, its noise-suppressing AGF combined with CBAM attention mechanisms effectively mitigates interference from complex backgrounds and illumination noise, while the unique uncertainty-aware mechanism enhances prediction confidence for dynamically quantifiable image regions. This approach not only precisely localizes hard-to-distinguish areas but also substantially improves pseudo-label reliability. At the merely 10% labeling ratio, where baseline models exhibit reduced stability due to insufficient supervision, introducing significant noise from prediction errors. USCount-Net's PLF mechanism which does not rely on labeled data for training, successfully filters noisy pseudo-labels. This results in a reduction of 3.93 in average MAE and 5.64 in average RMSE across phenological stages compared to the best-performing baseline (Calibrating), at 10% labeling ratio, collectively validating both the model architectural efficacy and its semi-supervised algorithm effectiveness.

Finally, Table S2 compares the proposed USCount-Net with other semi-supervised methods in terms of the number of parameters, floating-point operations per second (FLOPs), and inference time, which critically relevant to practical deployment. To ensure a fair comparison, the batch size was set to 1 for all methods on the self-constructed apple flower cluster dataset after preprocessing by CSAM-FE. The results indicate that USCount-Net achieves the second-best inference time. However, USCount-Net incurs higher FLOPs than IRAST and DREAM due to its greater architectural complexity. More significantly, this complexity is accompanied by a substantial improvement in counting performance, which aligns with the expectations. Overall, USCount-Net demonstrates competitive performance in terms of both model complexity and inference efficiency.

### 3.3 Ablation study

#### 3.3.1 Effect of the CSAM-FE

The CSAM-FE plays a critical role in facilitating subsequent fine-grained counting. By extracting floral clusters from extensive UAV imagery, this module provides purified inputs to downstream counting models with minimal background interference. To empirically validate its effectiveness, we conducted the ablation study on CSAM-FE using the original tree-level images, as summarized in Table 2. The results demonstrate a significant improvement in final flower counting accuracy, particularly for the bud and petal-fall flowers, whose small-sized targets are prone to being overwhelmed by background noise. And the tree-level inference time has been reduced by 0.69s.

**Table 2:** Ablation study of the CSAM-FE on the testing set. USCount-Net (w/o CSAM-FE) represents USCount-Net without CSAM-FE preprocessing; USCount-Net (w/CSAM-FE) represents the proposed full pipeline (CSAM-FE + USCount-Net).

Model	MAE			RMSE			Inference Times (s)
	Bud	Anthesis	Petal-Fall	Bud	Anthesis	Petal-Fall	
USCount-Net (w/o CSAM-FE)	50.66	15.32	38.12	110.28	34.53	62.77	3.16
USCount-Net (w/CSAM-FE)	**6.26**	**6.75**	**6.10**	**7.71**	**9.03**	**8.39**	**2.47**

**Fig. 6:**
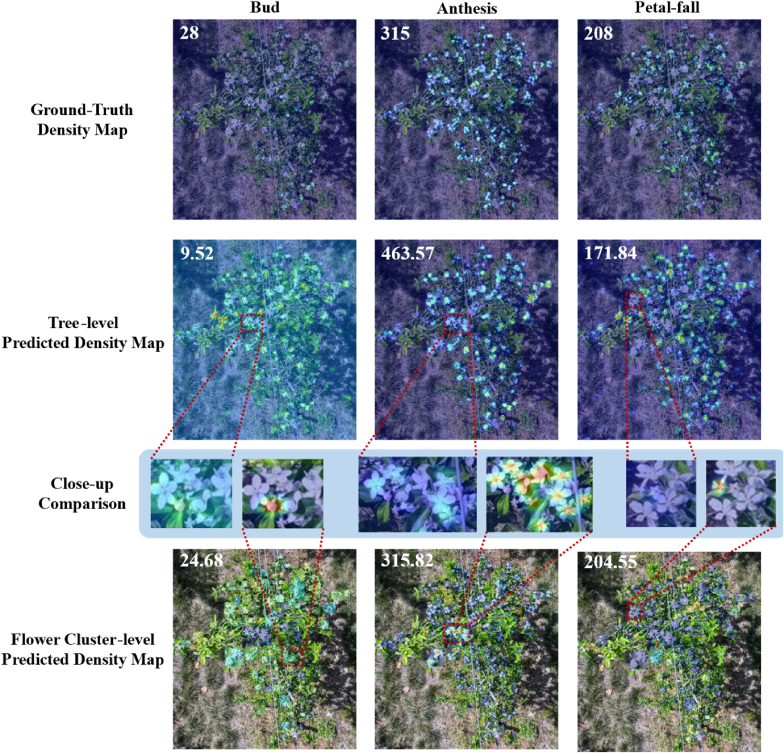
The visual comparison of predicted density map generated by USCount-Net with and without CSAM-FE. From top to bottom, the visualization displays: ground-truth density maps, predicted density maps from tree-level inputs, a close-up comparison between tree-level and flower cluster-level density maps, and predicted density maps from flower cluster-level inputs. Each set of density maps comprises representations for three flowering stages: bud, anthesis, and petal-fall, with the specific count values indicated in the top-left corner of each map.

Meantime, our comparison of density maps generated by USCount-Net with and without CSAM-FE intuitively reveals the effectiveness of CSAM-FE in reducing background noise interference and guiding model to focus on region of targets. Fig. 6 presents visualized density map predictions under two input scales for the highly challenging scenario of fruit trees during the late full-bloom stage. During this phenological phase, bud-stage flowers are extremely sparse, while anthesis and petal-fall stage apple flowers appear in abundant clusters.

The visualization comparison demonstrated that under tree-level input, the detection of bud-stage flowers nearly fails completely due to scarcity, while anthesis and petal-fall flowers exhibit significant missed detections and false positives owing to intra-cluster occlusion. In contrast, the flower cluster-level images extracted by CSAM-FE effectively forces the model to concentrate on local information, preventing small flower features from being overwhelmed by the extensive background. Consequently, this approach enables the model to more accurately capture the spatial distribution patterns of apple flowers across different phenological stages, ultimately achieving substantially improved accuracy in quantifying flowers throughout various phenological phases.

#### 3.3.2 Effect of the key components of USCount-Net

To validate the rationality and efficacy of the proposed model architecture, ablation studies were conducted under supervised (100% labeling ratio) and a representative semi-supervised setting (30% labeling ratio), as presented in Table 3. Specifically, the contributions of the AGF and CBAM modules to counting accuracy (Models 1-4) were evaluated under supervised, as they aim to enhance counting model performance. Meanwhile, the impact of the PLF mechanism (Models 5-6) was assessed under the 30% labeling ratio, since this strategy is specifically designed to boost consistency learning in low-label scenarios.

**Table 3:** Ablation study of the CBAM, AGF modules and PLF mechanism on the testing set. Results of our proposed models under both supervised and semi-supervised settings are in bold font.

Models	CBAM	AGF	PLF	Labeling Ratio	MAE			RMSE		
					Bud	Anthesis	Petal-fall	Bud	Anthesis	Petal-fall
1	×	×	—	100%	9.94	11.61	7.86	12.63	14.76	13.56
2	✓	×	—		8.37	10.84	8.83	10.94	13.72	11.37
3	×	✓	—		9.21	10.26	3.56	12.40	12.88	6.06
4	✓	✓	—		**6.26**	**6.75**	**6.10**	**7.71**	**9.03**	**8.39**
5	✓	✓	×	30%	8.03	9.81	4.89	11.73	14.40	7.99
6	✓	✓	✓		**7.26**	**8.90**	**6.41**	**10.78**	**13.16**	**9.33**

First, without the PLF mechanism, the impacts of AGF and CBAM modules on counting accuracy were analyzed. When introducing the CBAM module alone (Model 2), MAE and RMSE for bud and anthesis stages decreased, but MAE increased for the petal-fall stage. This could be attributed to the low-contrast characteristics of petal-fall images, where weak discriminability between target and background features. The CBAM attention weights, calculated based on local feature responses, amplify background noise interference in such scenarios. In contrast, when integrating the AGF module alone (Model 3), the counting errors across the three phenological stage decreased. This indicates that AGF dynamically allocates weights to preserve critical structural and semantic information in petal-fall stages while suppressing background noise.

Accordingly, a synergistic architecture combining AGF and CBAM (Model 4) was designed to leverage AGF's noise sensitivity for improved foreground-background discrimination. The results show that although the counting errors of petal-fall slightly increased compared to those of Model 3, the more critical task of counting bud and anthesis exhibited a marked reduction in errors, demonstrating enhanced performance through module collaboration.

Subsequently, the effect of the PLF mechanism in semi-supervised consistency regularization was evaluated at 30% labeling ratio. Results reveal that PLF substantially improves predictions for flowers at bud and anthesis stages but marginally reduces accuracy for petal-fall stage compared to Model 5 (without PLF). This phenomenon may arise from adaptive pseudo-label filtering, for flowers at petal-fall stage, where target-background discriminability is inherently low, the higher spatial uncertainty combined with strict filtering reduces effective supervision signals. However, despite small fluctuations in the accuracy of predictions on flowers in petal-fall stage, the significant improvements in predictions of critical bud and anthesis stages (vital for orchard management) make Model 6 the optimal overall solution.

### 3.4 Role of uncertainty estimation in enhancing semi-supervised learning

**Fig. 7:**
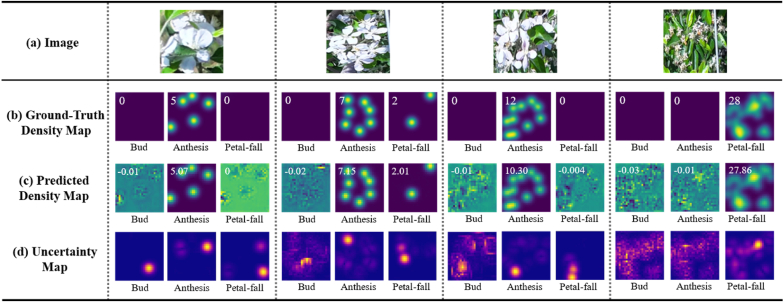
Visualization of predicted density maps and uncertainty maps in complex scenarios. (a) Input image; (b) Ground truth density maps for three phenological stages (Bud, Anthesis, Petal-fall) with count values annotated at the top-left corner; (c) Predicted density maps corresponding to each phenological stage with predicted count values annotated at the top-left corner; (d) Uncertainty maps corresponding to each phenological stage.

The proposed FFUE-based uncertainty quantification mechanism effectively assists the model in localizing challenging regions within complex scenes. As visualized in Fig. 7, uncertainty maps guide the model to precisely identify density estimation-sensitive areas caused by occlusion, high density, and scale variations, demonstrating robust counting performance.

To evaluate hyperparameter sensitivity, Table 4 presents counting errors analysis across phenological stages under varying forward propagation frequencies and uncertainty filtering threshold combinations. Notably, all analysis was conducted at the 30% labeling ratio, which serves as a representative for semi-supervised conditions. This setup maintains direct comparability among various hyperparameter configurations while ensuring the parameters are evaluated under the challenging low-label scenario they are designed to address.

**Table 4:** Performance comparison under varying uncertainty hyperparameters.

Forward Frequency	Threshold	MAE			RMSE		
		Bud	Anthesis	Petal-fall	Bud	Anthesis	Petal-fall
5	0.1	12.66	7.76	4.34	16.85	10.70	6.76
5	0.2	8.57	10.71	4.68	12.30	14.34	8.09
5	0.3	9.40	11.95	6.02	12.64	18.51	8.17
5	0.4	11.05	7.94	5.09	14.40	11.73	6.92

3	Adaptive Threshold	7.17	8.02	6.89	9.14	13.36	9.87
5	Adaptive Threshold	7.26	8.90	6.41	10.78	13.16	9.33
8	Adaptive Threshold	7.65	8.32	6.75	8.34	12.76	9.35
10	Adaptive Threshold	7.57	8.59	7.04	8.57	12.38	9.76

When fixing thresholds, forward passes were set to 5 to evaluate threshold sensitivity. As indicated in Table 4 (rows 1-4), model errors exhibit non-monotonic responses to threshold variations, suggesting that excessively high or low fixed thresholds may induce performance fluctuations. This indicates the inadequacy of fixed thresholds for pseudo-label filtering. In contrast, introducing an adaptive threshold mechanism significantly reduces model errors. Subsequently, the forward propagation frequency was validated under the adaptive threshold setting (rows 5-8). The results demonstrate that model performance remains stable across different frequencies, suggesting insensitivity to this parameter.

**Fig. 8:**
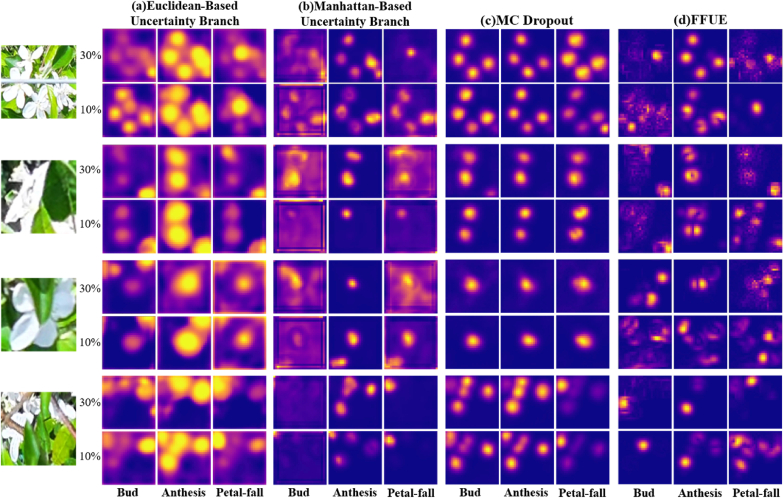
Visual comparison of uncertainty maps via different uncertainty measurement methods.
(2) MC Dropout [47]: Estimating uncertainty via stochastic dropout sampling (i.e., randomly discard neurons) during model inference (Fig. 8(c)).

Furthermore, the proposed FFUE quantifies spatial reliability through pixel-wise measurement of pseudo-label stability. By computing variance across multiple forward propagations, this method generates spatial uncertainty maps (Fig. 3(b)), requiring no additional supervision or architectural modifications. To validate its effectiveness in semi-supervised apple flower counting, two classical uncertainty estimation approaches were selected for comparison:

(1) Auxiliary uncertainty branch quantification [46]: Constructing an auxiliary branch to predict uncertainty maps and trains using similarity indices (Euclidean Distance in Fig. 8(a) and Manhattan Distance in Fig. 8(b)) between predicted and ground-truth density maps as supervisory signal.

Fig. 8 presents four representative instances corresponding to key apple flower counting challenges: dense distributions, severe occlusion, and morphological diversity. While all methods highlight uncertain regions (e.g., occluded or complex-background areas), the proposed FFUE (Fig. 8(d)) demonstrates superior sensitivity to boundary uncertainties between flowers and background interferences in complex foliage scenarios, capturing fine-grained discriminative details.

### 3.5 Generalization of the USCount-Net

To evaluate the generalization capability of the proposed method, validation was conducted on two public datasets: the screened and adapted apple flower dataset AriAplBud [48], and the extensively researched crowd counting dataset ShanghaiTech A [40]. Specifically, it focused on evaluating the robustness of the model architecture and its uncertainty-guided semi-supervised strategy.

AriAplBud is a specialized dataset for apple flower detection. It captures flowers at multiple developmental stages through close-range orthophotography of fruit tree. A total of 841 images closely relevant to this study were selected, constituting a dataset featuring the co-occurrence of apple flowers at multiple developmental stages, with 589 images allocated for training and 252 for testing. The screened dataset includes annotations of 7381 bud-stage flowers, 31193 anthesis-stage, and 4229 petal-fall-stage. It is noted that the annotation format was adapted by converting bounding box annotations to point annotations using box centroids, with density maps generated as final labels. This dataset evaluates USCount-Net's generalization capability for apple flower counting under varying imaging and geographical conditions.

As shown in Table S3, the results of comparison with state-of-the-art models on AriAplBud demonstrate that USCount-Net maintains superior performance across both supervised and semi-supervised settings, outperforming to other models across all three flower stages. It confirms the strong generalization capability of the proposed model for apple flower counting under diverse conditions.

ShanghaiTech A is a widely recognized benchmark crowd counting dataset, including 482 images with per-image annotations ranging from 33 to 3139. It is split into a training set of 300 images and a test set of 182 images. Following existing public partitioning protocol [49], the labeling ratios are set at 100% and 5%, 10%, 40% respectively for supervised and semi-supervised settings. This crowd dataset validates the cross-scene adaptability of USCount-Net.

As shown in Table S4, the results on ShanghaiTech A demonstrate that the proposed USCount-Net achieves competitive performance on crowd scene. While USCount-Net does not achieve top performance, attribute to its architecture being optimized for the non-uniform density distribution characteristic of apple flower scenes, which limits its adaptability to extremely dense crowd scenes. Nevertheless, it still maintains competitive performance compared to mainstream methods, particularly in supervised settings and semi-supervised settings with labeling ratios of 10% and 40%. This indicating its cross-scene adaptability.

As shown in Fig. S2 and Fig. S3, the USCount-Net effectively captures apple flower across multiple development stage and crowd density distribution. More, the uncertainty maps accurately identify challenging regions in dense scenes. This demonstrates that guided by the PLF mechanism, the model can rapidly locate areas of high uncertainty, thereby optimizing the semi-supervised consistency learning process. This confirms the across scenes transferability of the PLF mechanism.

## 4 Discussion

### 4.1 Innovations and performance analysis of the proposed framework

The proposed novel counting framework achieves accurate, phenological stage-specific apple flower counting from a UAV perspective, while significantly reduced annotation dependency. Notably, this study fills the research gap in previous studies that relied solely on side-view close-range photography for flower monitoring (e.g., YO-AFD [50], Chen et al. [10].) and lacked a high-throughput method to acquire the canopies flower density distribution of different phenological stage. While recent point cloud-based approach [51,52] provide valuable 3D structural insights, they require complex multi-view reconstruction, whereas our method achieves efficient canopy-level monitoring using single-view UAV imagery. And compared to the Density-Cluster-Count [15], which also focus on UAV-based apple flower density estimation but is limited to a single flowering period, this method provides more reliable information for orchard intelligent decision-making while reducing the model's reliance on large amounts of manually labeled data.

This capability to operate efficiently with minimal supervision while providing granular counts across bud, anthesis, and petal-fall stages stems from several synergistic innovations. First, the CSAM-FE employs a dual-stage strategy combining color threshold segmentation with SAM, significantly enhancing flower cluster extraction accuracy and providing high-quality input for the subsequent counting network. The core USCount-Net integrates several key components: the PLF mechanism quantifies pseudo-label uncertainty via FFUE, effectively suppressing low-quality pseudo-label interference even at low annotation rates, thereby enhancing robustness compared to existing semi-supervised models; AGF module dynamically fuses multi-scale features through weighted integration, balancing petal-level details with cluster-level semantic information.

Critically, at merely 30% annotation, the USCount-Net approaches supervised performance (Table 1). This demonstrates the effectiveness of the proposed architecture and semi-supervised training strategy in mitigating data annotation deficiencies.

This outcome stems from the dedicated noise-suppression components in the methodology. The crucial challenge of semi-supervised learning lies in generating reliable pseudo-labels from limited annotations and abundant unlabeled data. Background noise in input data (e.g., mislabeled samples or feature interference) degrades pseudo-label reliability, leading to model performance deterioration—aligning with Fooladgar [53] and Ortego [54]'s findings on “destructive impact of noise propagation on pseudo-label learning”. While prior works focus on improving model tolerance to low-quality pseudo-labels [46], this study introduces CSAM-FE, AGF, and PLF to suppress pseudo-label noise across three dimensions: input purification, prediction optimization, and pseudo-label filtering. Specifically, first of all, CSAM-FE eliminates the influence of background noise at preprocessing by leveraging color characteristics of flowers and the robust segmentation capability of SAM. Additionally, within USCount-Net, the AGF dynamically filters noise signals during the feature fusion, further reducing the impact of noise in the pseudo-label generation stage, while the PLF further weakens the influence of noisy pseudo-labels (which CSAM-FE and AGF have difficulty removing) in the post-processing stage of pseudo-labels on training.

Importantly, for objects that exhibit distinct disparities with background features, like apple flower, CSAM-FE achieves background noise removal at minimal cost through object-specific priors and the powerful zero-shot segmentation ability of SAM. This strategy holds broad applicability for dense small targets phenotyping.

### 4.2 Challenges of phenological stage-specific recognition

As shown in Fig. 1(c), petal-fall stage flowers exhibit tiny size and color similarity to the background, while the characteristics of UAV imaging further limit the acquisition of high-resolution images for this stage. These factors degrade feature discriminability, leading to performance degradation in both the PLF mechanism and CBAM module—components that perform well in bud and anthesis stages. Specifically, the weak feature distinctiveness of petal-fall flowers may cause the FFUE and CBAM modules to misidentify their features as noise, thereby compromising counting accuracy. To address this, we designed the noise-sensitive AGF module, which suppresses noise through nonlinear feature transformation via a gating unit while fusing multi-scale features. However, even with AGF integration, Petal-fall counting accuracy still shows a minor decline (Table 3). To mitigate this limitation, future work could incorporate multi-spectral data (e.g., thermal infrared bands) into RGB imagery, enabling the AGF module to weight-fuse visible and thermal features as auxiliary cues for better differentiation between Petal-fall flowers and the background.

From a practical orchard management perspective, bud stage flower counts determine initial fruit-set potential assessments, while anthesis stage density distributions are critical for predicting peak blooming periods. In contrast, petal-fall counts primarily serve phenological completeness documentation. Therefore, under resource constraints, we advocate prioritizing bud/and anthesis counting accuracy, which is vital for thinning decisions, while tolerating moderate petal-fall performance fluctuations.

### 4.3 Limitations of generalization

The color threshold-based prompt generation strategy in CSAM-FE is highly sensitive to imaging conditions and threshold settings. While the current thresholds are calibrated for canopy-level apple flowers under normal illumination, they may not generalize well under significant lighting variations or other real-world shifts, as shown in Fig. S4. To address this limitation, future work could explore adaptive thresholding strategies or utilize multi-channel color spaces in more variable environments, thereby enhancing the method's generalization capability under more diverse conditions.

Moreover, this study utilizes orchard imagery captured from flat plantations with fixed UAV shooting height and viewing angle. Although the dataset includes fruit trees of different cultivars and rootstock types, and the model's robustness has been validated on an additional public apple flower dataset, demonstrating its generalization capacity within similar domains, the validation in practical and diverse environments is still lacking. To address these limitations, future work will focus on two key dimensions. First, at the data level, a diversified data collection protocol including varied illumination intensities, geographic locations, shooting angles, and imaging devices, thereby augmenting the diversity of datasets to better reflect on-site complexity. Second, at the algorithmic level, future work could focus on constructing a cross-species flower feature bank through a meta-learning framework [55], enabling the model to learn generic feature representations. Alternatively, generative adversarial nets (GAN) [56] or diffusion models [57] could be employed to synthesize varied images, expanding counting robustness to the variation of flower morphology and environment through data augmentation.

### 4.4 Potential of practical deployment

In terms of practical application, integrating this method into intelligent orchard systems to provide canopy-level flower density distribution maps during the flowering stage can offer critical spatial information for flower thinning decisions. Studies have demonstrated that vision-based flower density assessment is viable for guiding flower thinning operations [11]. While obtaining complete, flower-level phenological information for individual trees remains a challenge for automated flower thinning at the current stage, the canopy-level, stage-specific flower cluster density assessment provided by the proposed framework already holds practical value for guiding preliminary decision-making in chemical or mechanical flower thinning.

Furthermore, this work enables to achieve accurate distribution and quantification of apple flowers across three key flowering stages: bud, anthesis, and petal-fall. Even without direct integration into real-time flower thinning equipment currently, the proposed framework establishes a quantitative link between perceptual data and management decisions, laying a necessary foundation for future closed-loop systems that connect sensing, optimization, and execution.

Future research could explore the practical deployment of high-precision algorithms within intelligent orchard systems, constructing an end-to-end “lightweight sensing – model prediction – decision execution” closed-loop architecture, ultimately achieving precise, resource-efficient, and automated orchard management.

## 5 Conclusion

To address the challenges of scarce annotation data, background noise interference, and significant scale variations in apple flower counting from UAV images in agricultural scenarios, an uncertainty-guided semi-supervised flower counting model (USCount-Net) is proposed. The model significantly reduces annotation costs while enhancing robustness in complex environments, offering an efficient solution for separately counting apple flower at various phenological stages in challenging conditions. The key contributions and conclusions are as follows: (1) The training-free CSAM-FE preprocessing module, which synergizes color thresholding with SAM to boost flower cluster extraction accuracy in cluttered backgrounds without additional cost. This approach preserves target integrity while compressing processing data by >90%, simultaneously improving counting precision and reducing model computational complexity; (2) The PLF mechanism, leveraging FFUE to quantify pseudo-label reliability and dynamically suppress noise via adaptive thresholds. At 10% annotation, USCount-Net reduces average MAE and RMSE by 3.93 and 5.64 versus state-of-the-art semi-supervised models, effectively mitigating label degradation from illumination variance and occlusion.; (3) The AGF module, which enhances multi-scale perception through context-aware feature weighting. Ablation studies confirm that the synergistic integration of AGF with CBAM and PLF delivers optimal or near-optimal counting errors across all phenological stages, demonstrating the robustness of model to scale variations and environmental complexity. Together, these innovations enable USCount-Net to balance accuracy, efficiency, and adaptability in low-annotation agricultural scenarios.

Experimental results show that USCount-Net achieves counting accuracy comparable to supervised models at 10%-50% annotation rates on a self-built apple flower counting dataset, outperforming existing semi-supervised methods. This highlights its practical applicability, providing an efficient and low-cost solution for orchard flower-load assessment with significant implications for thinning decisions and yield prediction. Future work will focus on improving cross-species generalization capabilities and optimizing the computational efficiency of the uncertainty estimation module to meet real-time operational demands in field environments.

## Author contributions

Yu Wang constructed the model, conducted the experimental investigations, generated the figures, and drafted the original manuscript. Jingzhong Huang, Chengyu Chen, Xiangfei Zhuge, and Wen Chu performed data collection, data processing, and data labeling. Xia Hao and Xuchao Guo co-conceptualized the study, provided theoretical guidance, supervised the experiments, and undertook the critical review and revision of the manuscript.

## Declaration of competing interest

The authors declare that they have no known competing financial interests or personal relationships that could have appeared to influence the work reported in this paper.

## Data Availability

The source code is publicly available at https://github.com/haohuihui5019/USCount-Net. And the source dataset can be accessed at https://drive.google.com/drive/folders/1KP8H0qIuct56hWre5GV6ZJnzw qOY3Pip.
