# Supplementary material for: Low-annotation apple flower counting: A color-SAM enhanced and uncertainty-guided semi-supervised framework
Source: Plant Phenomics. 2026 Mar 3;8(2):100190. doi: 10.1016/j.plaphe.2026.100190 (PMC13316440; doi:10.1016/j.plaphe.2026.100190)
Supplement: Multimedia component 1 [file mmc1.docx]

# **Supplementary materials**


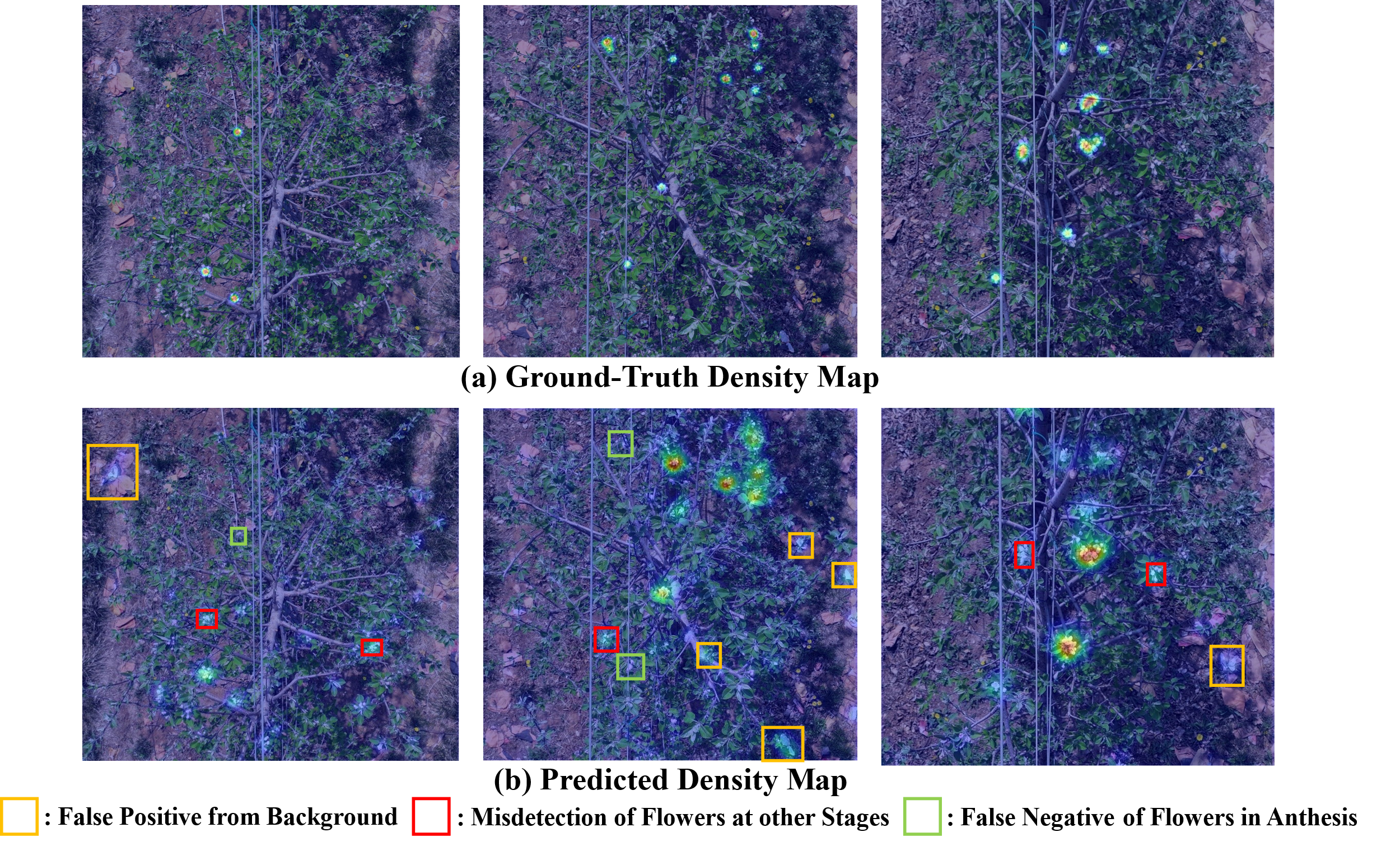


**Fig. S1.** Illustration of density estimation errors for anthesis apple flowers from UAV imagery


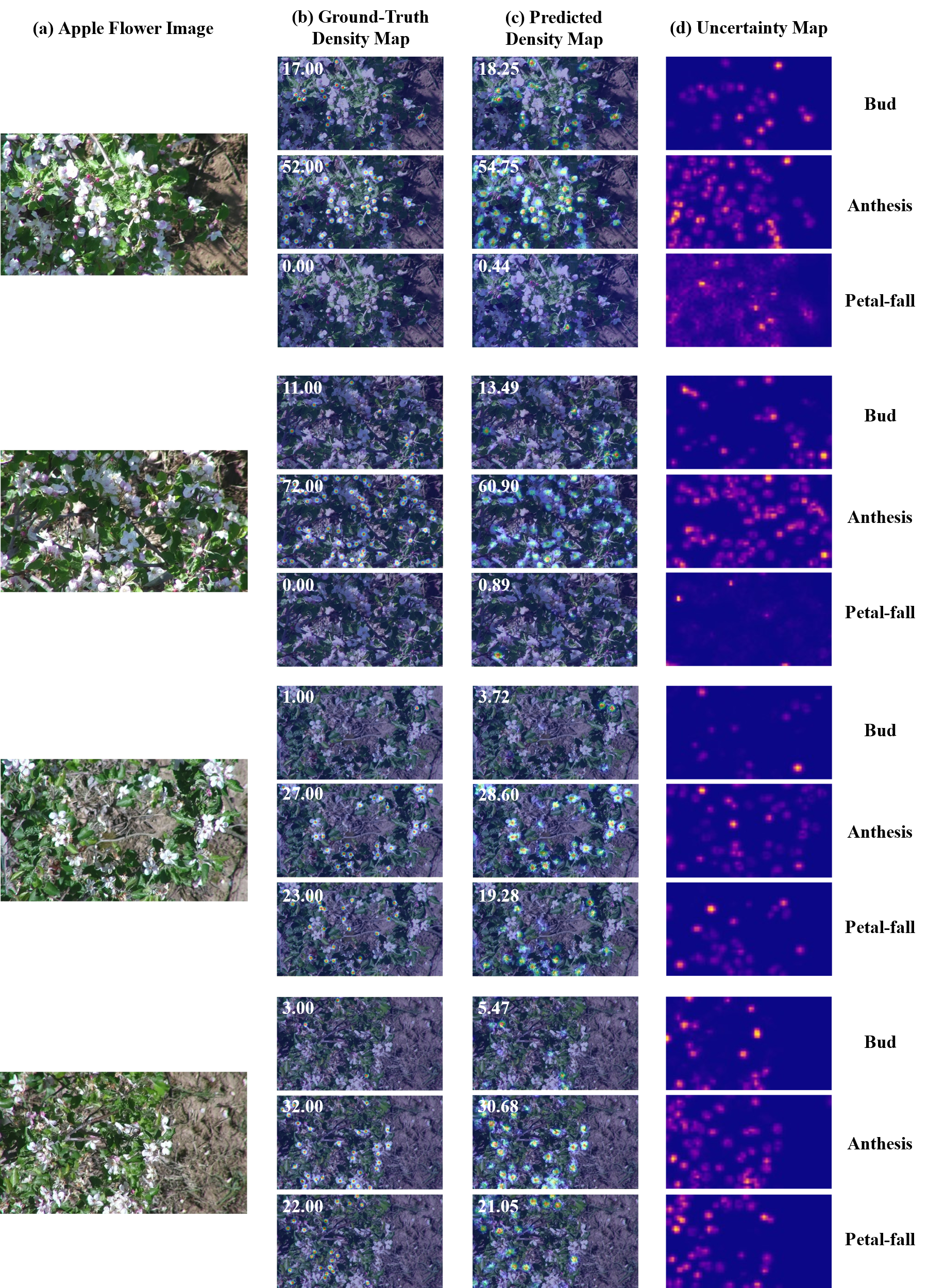


**Fig. S2.** Visualization of predicted density maps and uncertainty maps on AriAplBud. (a) Input image; (b) Ground truth density maps with true count values annotated at the top-left corner; (c) Predicted density maps with predicted count values annotated at the top-left corner; (d) Uncertainty maps indicating the prediction confidence in a certain region.


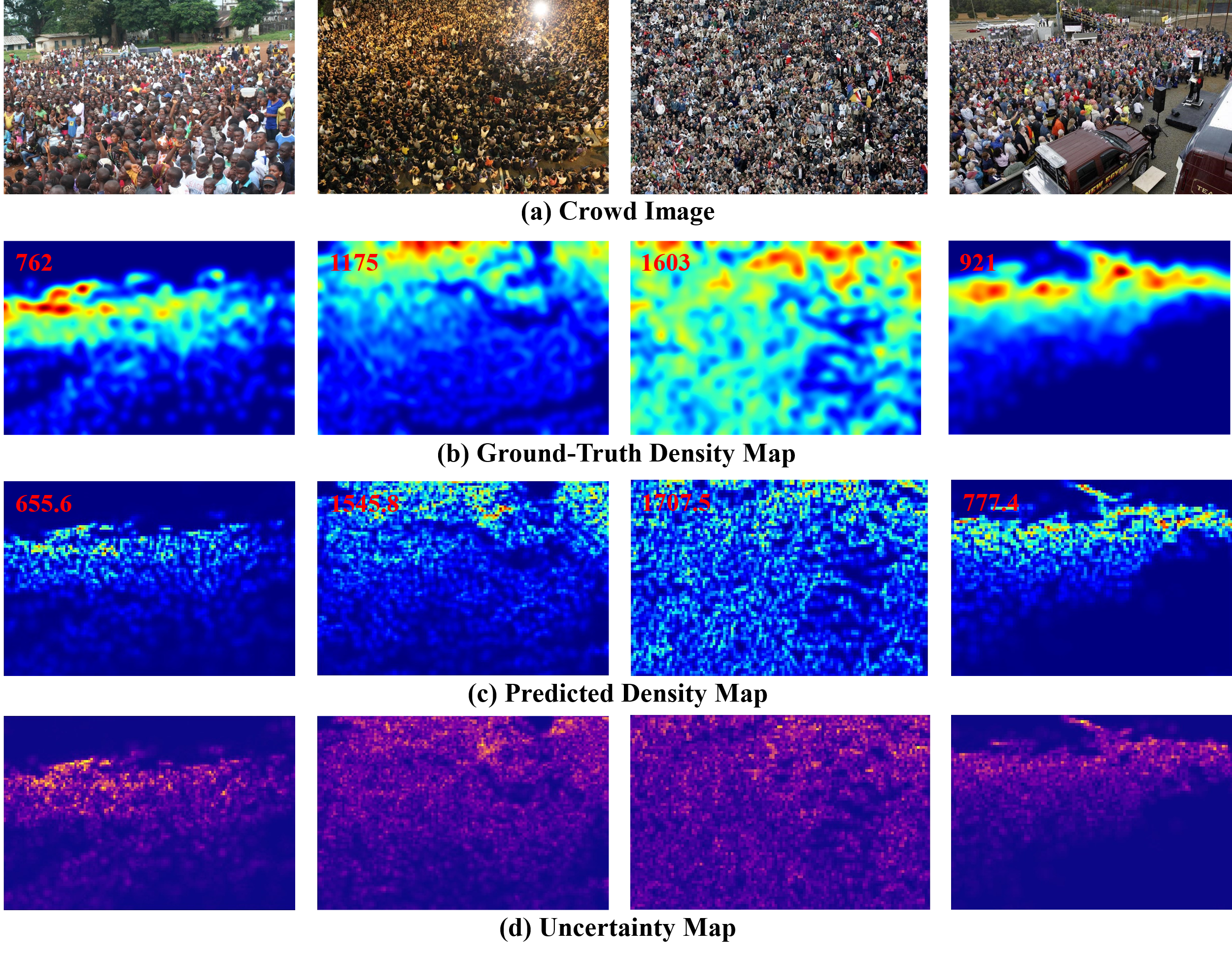


**Fig. S3.** Visualization of predicted density maps and uncertainty maps on ShanghaiTech A. (a) Input image; (b) Ground truth density maps with true count values annotated at the top-left corner; (c) Predicted density maps with predicted count values annotated at the top-left corner; (d) Uncertainty maps indicating the prediction confidence in a certain region.


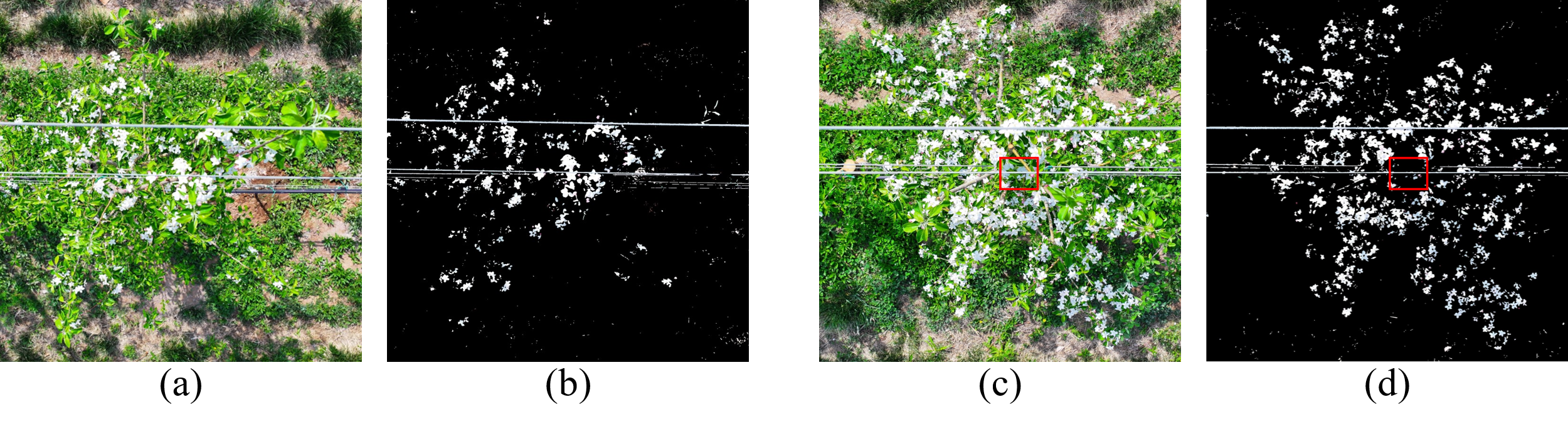


**Fig. S4.** Impact of intense variations in illumination on the color threshold-based prompt generation. (a) and (c) showcase original images of apple trees, whereas (b) and (d) illustrate the results of color threshold pixel extraction. The red box indicates a sample region where pixel extraction failed due to excessively dark flower clusters.

**Table S1.** Environment configuration and experiment details.

| Hardware | Configuration | Software | Version | Training Parameters | Value |
| --- | --- | --- | --- | --- | --- |
| CPU | Intel Core i9-12900K | PyTorch | 1.13.1 | Learning Rate | 1e-5 |
| RAM | 64 GB DDR5 | Python | 3.8 | Batch Size | 16 |
| GPU | NVIDIA RTX 3090 (24GB) | CUDA | 11.8 | Optimizer | Adam |
| OS | Windows 11 | PyCharm | 2023.1.2 | Epochs | 200 |

**Table S2.** Comparison of deployment efficiency metrics with other counting models on the testing set.

| Model | FLOPs (G) | Number of Parameters (M) | Inference Times (ms) |
| --- | --- | --- | --- |
| MT | 20.32 | 15.91 | 3.26 |
| IRAST | 21.59 | 17.37 | 4.26 |
| DREAM | 20.73 | 16.26 | 3.55 |
| TreeFormer | 27.83 | 141.38 | 15.59 |
| Calibrating | 108.24 | 24.62 | 5.27 |
| USCount-Net (Ours) | 26.90 | 35.39 | 4.13 |

**Table S3.** Comparisons With the State-of-the-Art Methods on AriAplBud. The best results are in bold font, and the second-best results are underlined.

| Method | Model | Labeling  Ratio |  | MAE | | |  | RMSE | | |
| --- | --- | --- | --- | --- | --- | --- | --- | --- | --- | --- |
|  |  |  |  | Bud | Anthesis | Petal-fall |  | Bud | Anthesis | Petal-fall |
| Supervised | MCNN | 100% |  | 6.85 | 12.65 | 10.47 |  | 8.54 | 18.27 | 14.53 |
|  | CSR-Net |  |  | 3.42 | 5.32 | 3.87 |  | 3.21 | 6.54 | 4.65 |
|  | DM-Count |  |  | 3.18 | 6.24 | **2.42** |  | 3.94 | 8.32 | 3.63 |
|  | **USCount-Net** |  |  | **2.42** | **4.89** | 2.67 |  | **3.21** | **6.05** | **3.57** |
| Semi-Supervised | MT | 10% |  | 8.95 | 10.74 | 12.16 |  | 12.25 | 14.32 | 16.07 |
|  | IRAST |  |  | 5.64 | 9.83 | 10.47 |  | 7.70 | 12.40 | 14.87 |
|  | Dream |  |  | 17.37 | **4.34** | 24.08 |  | 20.52 | **5.24** | 27.19 |
|  | TreeFormer |  |  | 11.06 | 23.84 | 26.65 |  | 11.25 | 24.80 | 26.98 |
|  | Calibrating |  |  | 5.32 | 7.84 | 5.67 |  | 6.65 | 9.46 | 7.63 |
|  | **USCount-Net** |  |  | **2.91** | 6.44 | **3.23** |  | **3.85** | 8.37 | **4.60** |
|  | MT | 30% |  | 7.84 | 8.68 | 10.50 |  | 11.13 | 11.85 | 13.49 |
|  | IRAST |  |  | 4.95 | 7.20 | 7.89 |  | 6.50 | 8.93 | 10.66 |
|  | Dream |  |  | 14.57 | **4.13** | 15.10 |  | 19.64 | **5.01** | 19.78 |
|  | TreeFormer |  |  | 8.83 | 15.36 | 18.57 |  | 11.26 | 20.62 | 22.74 |
|  | Calibrating |  |  | 4.54 | 6.92 | 5.72 |  | 6.37 | 8.63 | 7.85 |
|  | **USCount-Net** |  |  | **3.50** | 5.37 | **2.16** |  | **4.63** | 7.19 | **3.37** |
|  | MT | 50% |  | 6.73 | 7.29 | 9.63 |  | 8.72 | 9.06 | 12.87 |
|  | IRAST |  |  | 4.17 | 6.97 | 7.54 |  | 5.71 | 8.79 | 9.25 |
|  | Dream |  |  | 8.46 | **4.05** | 9.72 |  | 13.58 | **5.14** | 12.72 |
|  | TreeFormer |  |  | 7.26 | 13.54 | 16.83 |  | 9.87 | 18.52 | 21.23 |
|  | Calibrating |  |  | 4.31 | 6.20 | 5.34 |  | 5.85 | 8.03 | 6.61 |
|  | **USCount-Net** |  |  | **2.83** | 5.99 | **2.63** |  | **3.68** | 7.77 | **3.66** |

**Table S4.** Comparisons With the State-of-the-Art Methods on ShanghaiTech A. The results from other methods are reported as in the original papers. The best results are in bold font, and the second-best results are underlined.

| Method | Model | Venue | Labeling Ratio | MAE | RMSE |
| --- | --- | --- | --- | --- | --- |
| Supervised | MCNN | NeurlPS'17 | 100% | 110.2 | 173.2 |
|  | CSR Net | CVPR'18 |  | 68.2 | 115.0 |
|  | DM-Count | NeurlPS'20 |  | **59.7** | **95.7** |
|  | USCount-Net (Ours) | — |  | 63.21 | 101.67 |
| Semi-supervised | MT | NeurlPS'17 | 5% | 104.7 | 156.9 |
|  | L2R | CVPR'18 |  | 103.0 | 155.4 |
|  | OT-M | CVPR'23 |  | **83.7** | **133.3** |
|  | Dream | TNNLS'25 |  | 112.7 | 165.7 |
|  | USCount-Net (Ours) | — |  | 107.5 | 150.6 |
|  | MT | NeurlPS'17 | 10% | 94.5 | 156.1 |
|  | L2R | CVPR'18 |  | 90.3 | 153.5 |
|  | IRAST | ECCV'20 |  | 86.9 | 148.9 |
|  | OT-M | CVPR'23 |  | **80.1** | **118.5** |
|  | Dream | TNNLS'25 |  | 112.7 | 165.7 |
|  | USCount-Net (Ours) | — |  | 83.4 | 144.6 |
|  | MT | NeurlPS'17 | 40% | 88.2 | 151.1 |
|  | L2R | CVPR'18 |  | 86.5 | 148.2 |
|  | Calibrating | ICCV'23 |  | 70.8 | 116.6 |
|  | OT-M | CVPR'23 |  | 70.7 | 114.5 |
|  | Dream | TNNLS'25 |  | 78.4 | 112.9 |
|  | USCount-Net (Ours) | — |  | **70.0** | **115.3** |
